# YKL-39 is an independent prognostic factor in gastric adenocarcinoma and is associated with tumor-associated macrophage infiltration and angiogenesis

**DOI:** 10.1186/s12957-022-02830-9

**Published:** 2022-11-14

**Authors:** Ling Xue, Wei Chu, Fangsheng Wan, Pingfan Wu, Xiaowen Zhao, Linna Ma, Yali She, Changtian Li, Yaling Li

**Affiliations:** 1 College of Basic Medicine, Gansu University of Chinese Medicine, Lanzhou, 730000 China; 2 Department of Pathology, the 940th Hospital of the Joint Logistic Support of the People’s Liberation Army, Lanzhou, 730050 China; 3 Provincial-Level Key Laboratory of Molecular Medicine of Major Diseases and Study on Prevention and Treatment of Traditional Chinese Medicine, Gansu University of Chinese Medicine, Lanzhou, Gansu China

**Keywords:** Gastric cancer, Tumor-associated macrophage, Tumor angiogenic, Prognosis

## Abstract

**Background:**

Gastric cancer has a high incidence and mortality rate. Angiogenesis is necessary for tumor infiltration and metastasis and affects patient prognosis. YKL-39 has monocyte chemotactic activity and pro-angiogenic activity in some tumors. In this study, we investigated the relationship between YKL-39 and tumor-associated macrophages and microangiogenesis in gastric cancer to determine its potential as a prognostic biomarker.

**Materials and methods:**

A total of 119 patients with gastric cancer who had undergone gastrectomy at the 940th Hospital of the Joint Security Force between 2014 and 2018 were included in this study. We assayed the protein expression of YKL-39, CD68, and CD34 by immunohistochemistry in tissues of 119 patients with gastric cancer, as well as the intracellular expression of YKL-39 and CD68 by immunofluorescence. Data were analyzed with SPSS Statistics 25.0 to explore the impact of expression of YKL-39, CD68, and CD34 in gastric cancer patients and the relationship among them.

**Results:**

Our results show that YKL-39 was expressed in both the nucleus and cytoplasm of gastric cancer cells and tumor mesenchyme. YKL-39 protein expression was associated with the depth of tumor infiltration, lymph node metastasis, and TNM stage; CD68 protein expression was associated with lymph node metastasis and TNM stage; CD34 protein expression was not associated with clinicopathological characteristics. Expression of YKL-39 was positively correlated with CD68 and CD34 (*p* < 0.001), and high expression of YKL-39 was associated with poor prognosis (*p* < 0.05).

**Conclusion:**

In gastric cancer, YKL-39 expression is positively correlated with the degree of tumor-associated macrophage infiltration and angiogenesis, and is a potential prognostic marker for gastric cancer.

## Introduction

Gastric cancer (GC) is one of the most common gastrointestinal malignancies, ranking fifth in incidence and fourth in lethality globally according to the latest global cancer statistics [1]. With advances in technology such as endoscopy, an increasing number of patients are being diagnosed with GC and treated in a timely manner, but the overall 5-year survival rate is still less than 40% [2]. Most GC patients are already in the middle or late stages when they are diagnosed, with a median overall survival (OS) of less than 12 months [3]. Angiogenesis supports the growth and metastasis of GC cells by providing nutrients and oxygen [4]; when the tumor has advanced metastasis or cannot be excised, the current treatment is based on palliative chemotherapy, and anti-angiogenic therapy can be used as an effective adjuvant treatment [5]. The commonly used anti-angiogenic drugs include bevacizumab, which targets vascular endothelial growth factor-A (VEGF-A), ramucirumab, which targets vascular endothelial growth factor receptor 2, and ziv-aflibercept, which targets VEGF-A isoforms, placental growth factor (PLGF), and vascular endothelial growth factor-B etc .[6]. However, tumors can develop resistance to anti-angiogenic drugs through a variety of mechanisms, including upregulation of alternative pro-angiogenic signaling pathways, resistance of tumor stromal cells to anti-angiogenic drugs, adaptation of tumor cells to hypoxic environments, and alternative mechanisms of tumor vascularization [7]. Therefore, it is of great significance to find new anti-angiogenic targets.

Tumor-associated macrophages (TAM) are the most common tumor-infiltrating immune cells in the tumor microenvironment (TME), accounting for more than 50% of immune cells in the TME and promoting tumorigenesis through various mechanisms such as stimulating angiogenesis, increasing tumor cell invasion and migration, and inhibiting anti-tumor immunity [8, 9]. Macrophages are stimulated by different chemokines released by tumors and stromal cells to differentiate into two phenotypes with dramatic differences: M1 macrophages with antitumor effects and M2 macrophages with pro-tumor effects [8]. M2 macrophages, which occupy the majority of TAM, can produce a variety of pro-angiogenic factors such as VEGF-A and tumor necrosis factor α (TNFα) in hypoxic areas to maintain tumor growth [10]. TAM infiltration in multiple tumors is positively correlated with angiogenesis [11, 12], and it has been shown that the emergence of anti-VEGF therapy resistance is associated with the aggregation of TAMs in TME [13, 14].

Chitinase 3-like protein 2(CHI3L2), also known as YKL-39, belongs to the family of chitinase-like proteins (CLPs) that function as both cytokines and growth factors [15]. Human CLPs include chitinase 3-like protein 1 (YKL-40), YKL-39 and stabilin-1 interacting chitinase-like protein (SI-CLP). YKL-39 was originally found in human synoviocytes and chondrocytes and plays a role in regulating autoimmunity and participating in tissue remodeling [16]. It has been shown that YKL-39 expression is elevated in degenerative pathologies and diseases characterized by tissue remodeling, such as osteoarthritis, multiple sclerosis, Alzheimer’s disease, and amyotrophic lateral sclerosis [17–19]. Recent studies have reported that YKL-39 has monocyte chemotactic and pro-angiogenic activity and is expressed in M2 macrophages from breast, glioma, and kidney cancers, and affects tumor angiogenesis; overexpressed YKL-39 is associated with poor prognosis [20–22]. However, there are no reports on the relationship between YKL-39 expression and GC biological behavior and the prognosis of GC patients.

CD68 is a highly glycosylated transmembrane protein associated with lysosomal particles and is currently the most widely used marker for immunohistochemical recognition of macrophages [23]. CD68 is a major biomarker for detecting total TAMs in a variety of malignant cancers including breast, colorectal, lung, ovarian, and lymphoma [24]. The commonly used vascular markers are CD31, CD34, and factor VIII, among which CD31 mainly marks neovascularization and CD34 marks all vascular endothelium including neovascularization and mature vessels [25].

In this study, we used immunohistochemistry (IHC) and immunofluorescence (IF) to detect the expression of YKL-39, CD68, and CD34 in GC tissues to determine the relationship between YKL-39 expression and macrophage infiltration and angiogenesis.

## Material and methods

### Patients and tissue samples

This study included 119 patients diagnosed with GC through histopathologic evaluation on gastroscopic biopsy or surgical tissue specimens; 101 cases were male and 18 cases were female, ranging in age from 29 to 81 years, with a median age of 59.03 years. All patients underwent gastrectomy between 2014 and 2018 at the 940th Hospital of the Joint Security Force, Gansu, China. Patients who had preoperative treatment, such as radiotherapy, chemotherapy, or other medical interventions and those diagnosed with autoimmune diseases were excluded from the study. Formalin-fixed, paraffin-embedded (FFPE) GC tissue samples were obtained from these patients after surgery.

### Data collection and follow-up

Demographic and clinicopathologic characteristics, including age, sex, TNM stage, histologic grade, and lymph node metastases were assessed. The TNM classification was based on the 8th edition of AJCC GC staging. The patients were followed up for 5 years by inpatient, outpatient and telephone contact. In this study, we used OS to evaluate prognosis. OS was defined as the period from the date of surgery to the date of death or last visit. The median follow-up time was 44.18 months (range 3–114 months). Informed consent was obtained from all patients.

### Tissue microarray (TMA) construction

FFPE specimens were stained with H&E and examined by experienced pathologists. At least three representative regions were selected and marked on H&E slices each tumor. The corresponding part on the wax block was selected as the material selection site according to the mark on the H&E slice. TMAs containing the 119 FFPE specimens were constructed by using a Manual Tissue Arrayer (TM1, boyikang, Bejing, China). All three marked regions on each tissue with a diameter of 2.0 mm were removed using a TMA instrument and placed into the acceptor wax block from top to bottom in a right-to-left order.

### IHC

All TMA slides with a thickness of 4 μm were deparaffinized using xylene and rehydrated using graded ethanol. Then, the slides were immersed and boiled in ethylene diamine tetraacetic acid (pH 9.0) for 30 min in a pressure cooker for antigen retrieval. Endogenous peroxidase was inhibited by 3% H_2_O_2_ for 15 min. For YKL-39 staining, recombinant monoclonal rabbit anti-human YKL-39 (1:150; ThermoFisher, USA) was applied. For TAM evaluation, monoclonal mouse anti-human CD68 (ready-to-use; maixin, China) was applied. For micro vessel density (MVD) evaluation, monoclonal mouse anti-human CD34 (ready-to-use, maixin, China) was used. Specimens were incubated with all antibodies overnight at 4 °C. After washing with PBS, the slides were incubated with secondary antibody at 37 °C for 10 min. The following steps were performed: color development, counterstaining, differentiation, dehydration, and transparency. Finally, the slides fixed with neutral resin.

IHC slices were examined at a low magnification (× 100) and the most representative five high-magnification (× 400) fields were selected for staining assessment. A semi-quantitative IHC scoring criterion was used to determine the YKL-39 protein expression levels in tumor. The percent positivity of staining cells ranged from 0 to 4: 0, none; 1, 1–25%; 2, 26–50%; 3, 51–75%; 4, 76–100%. The intensity of staining was graded from 0 to 3 (0, no staining; 1, weak; 2, moderate; and 3, strong). We obtained the final IHC score by multiplying the proportion score by the intensity score. A median score of 6 (> 6 or ≤ 6) was selected as the cutoff to distinguish patients with YKL-39-positive or YKL-39-negative expression. CD68 expression was determined by the number of positive cells expressed, not the intensity of staining. The proportion of cells staining positively for CD68 to tumor interstitial cells was calculated as the CD68-positive rate which was used as a score, ranging from 0 to 10. By counting the staining results and calculating the median, it was determined that 6 was the cutoff point to distinguish between high and low expression of CD68, with a score ≥ 6 for high expression and < 6 for low expression [20]. The number of CD34-positive labeled vessels was determined with each vessel counted as one point. Five × 200 fields of view were observed and the average value was calculated. A median of 17.7 was calculated by counting the staining results to distinguish between dense and sparse angiogenesis [26].

### IF staining and confocal microscopy

Formalin fixed, paraffin embedded sections were deparaffinized by xylene and rehydrated. The slices were placed in a pressure cooker with sodium citrate and boiled, then cooled gradually to room temperature to complete the antigen repair. Blocking solution consisted of 0.3% Triton + 10 mg/mL bovine serum albumin + PBS and used for 1 h. The primary antibodies used include YKL-39 (1:100, Bioss, China) and CD68 (1:50, BOSTER, China) and were incubated with the tissue overnight at 4 °C. The secondary antibodies used were goat anti-mouse-Alexa Fluor 594 (1:200) and goat anti-rabbit-Alexa Fluor 488 (1:200) and were used according to instructions. The secondary antibodies were dropped onto the tissues and incubated for 2 h at room temperature and protected from light. After cleaning the slides with PBS dilution, anti-fluorescence quenching sealing liquid (including DAPI) was added and the slides were stored at 4 °C and protected from light.

Confocal laser scanning microscopy was performed with a Leica TCS SP8 laser scanning spectral confocal microscope (OLYMPUS, FV10-ASW2.1 Viewer, Japan), equipped with a 63 £ 1.32 objective. Excitation was with laser emitting at 405 nm, 488 nm, and 559 nm. All two-or three-color images were acquired using a sequential scan mode.

### Statistical analysis

SPSS 25.0 was used for statistical analysis. The relationship between YKL-39, CD68, CD34, and clinicopathological characteristics of patients was tested by a *χ*² test. Correlation analysis used Spearman's rank correlation coefficient. Survival analyses were conducted using the Kaplan-Meier method and differences in survival were examined using the log-rank test. Univariate and multivariate analyses were conducted using the Cox proportional hazards regression models. Statistical significance was defined as a value of *p* < 0.05.

## Results

### Relationship between YKL-39, CD68, and CD34 expression and clinicopathological characteristics of GC patients

Among the 119 IHC staining results, 69 (57.98%) cases showed elevated expression of YKL-39, 68 (57.14%) cases showed elevated expression of CD68, and 59 (49.58%) cases showed elevated expression of CD34.

We analyzed the correlation between YKL-39, CD68, and CD34 expression and patient gender, age, depth of tumor infiltration, lymph node metastasis, distant metastasis, TNM stage, and degree of differentiation. YKL-39 protein expression was associated with the depth of tumor infiltration (*p* = 0.018), lymph node metastasis (*p* = 0.029), and TNM stage (*p* = 0.003). Tumor infiltration was deeper, lymph node metastasis was more frequent, and TNM stage was higher, and YKL-39 expression was higher. CD68 protein expression was associated with lymph node metastasis (*p* = 0.048) and TNM stage (*p* = 0.029), with more lymph node metastasis and higher TNM stage associated with higher CD68 expression. There was no significant correlation between CD34 expression and clinicopathological characteristics (Table 1).

**Table 1:** Association of YKL-39, CD68, CD34 protein expression with clinical and pathological parameters (*n* = 119)

Clinical factors	total	Expression of YKL-39		*χ2*	*p* value	Expression of CD68		*χ2*	*p* value	Expression of CD34		*χ2*	*p* value
		Low (*n* = 50)	High (*n* = 69)			Low (*n* = 51)	High (*n* = 68)			Low (*n* = 60)	High (*n* = 59)		
Gender				0.555	0.456			1.31	0.252			2.239	0.135
Male	101(84.87)	41(34.45)	60(50.42)			46(38.66)	55(46.22)			48(40.34)	53(44.54)		
Female	18(15.13)	9(7.56)	9(7.56)			5(4.20)	13(10.92)			12(10.08)	6(5.04)		
Age (years)				0.125	0.724			1.754	0.185			1.011	0.315
> 60	57(47.90)	23(19.33)	34(28.57)			28(23.53)	29(24.37)			26(21.85)	31(26.05)		
≤ 60	62(52.10)	27(22.69)	35(29.41)			23(19.33)	39(32.77)			34(28.57)	28(23.53)		
Depth of tumor invasion				**5.602**	**0.018**			0.474	0.491			1.416	0.234
T1–T3	61(51.26)	32(26.89)	29(24.37)			28(23.53)	33(27.73)			34(28.57)	27(22.69)		
T4	58(48.74)	18(15.13)	40(33.61)			23(19.33)	35(29.41)			26(21.85)	32(26.89)		
Lymph node metastasis				**9.005**	**0.029**			**7.88**	**0.048**			5.312	0.154
N0	30(25.21)	19(15.97)	11(9.24)			18(15.13)	12(10.08)			19(15.97)	11(9.24)		
N1	25(21.01)	11(9.24)	14(11.76)			13(10.92)	12(10.08)			15(12.61)	10(8.40)		
N2	34(28.57)	12(10.08)	22(18.49)			11(9.24)	23(19.33)			14(11.76)	20(16.81)		
N3	30(25.21)	8(6.72)	22(18.49)			9(7.56)	21(17.65)			12(10.08)	18(15.13)		
Distant metastases				2.24	0.134			0.706	0.401			0.708	0.4
M0	79(66.39)	37(31.09)	42(35.29)			36(30.25)	43(36.13)			42(35.29)	37(31.09)		
M1	40(33.61)	27(22.69)	13(10.92)			15(12.61)	25(21.01)			18(15.13)	22(18.49)		
TNM stage				**14.148**	**0.003**			**8.898**	**0.029**			6.52	0.085
I	12(10.08)	11(9.24)	1(0.84)			10(8.40)	2(1.68)			10(8.40)	2(1.68)		
II	22(18.49)	9(7.56)	13(10.92)			9(7.56)	13(10.92)			12(10.08)	10(8.40)		
III	44(36.97)	17(14.29)	27(22.69)			16(13.45)	28(23.53)			20(16.81)	24(20.17)		
IV	40(33.61)	12(10.08)	28(23.53)			15(12.61)	25(21.01)			17(14.29)	23(19.33)		
Degree of differentiation				0.496	0.481			2.707	0.1			0.218	0.641
Well differentiation	55(46.22)	25(21.01)	30(25.21)			28(23.53)	27(22.69)			29(24.37)	26(21.85)		
Poor differentiation	64(53.78)	25(21.01)	39(32.77)			23(19.33)	41(34.45)			31(26.05)	33(27.73)		

### Correlation between YKL-39 expression and TAMs infiltration and angiogenesis in GC tissue

**Fig. 1:**
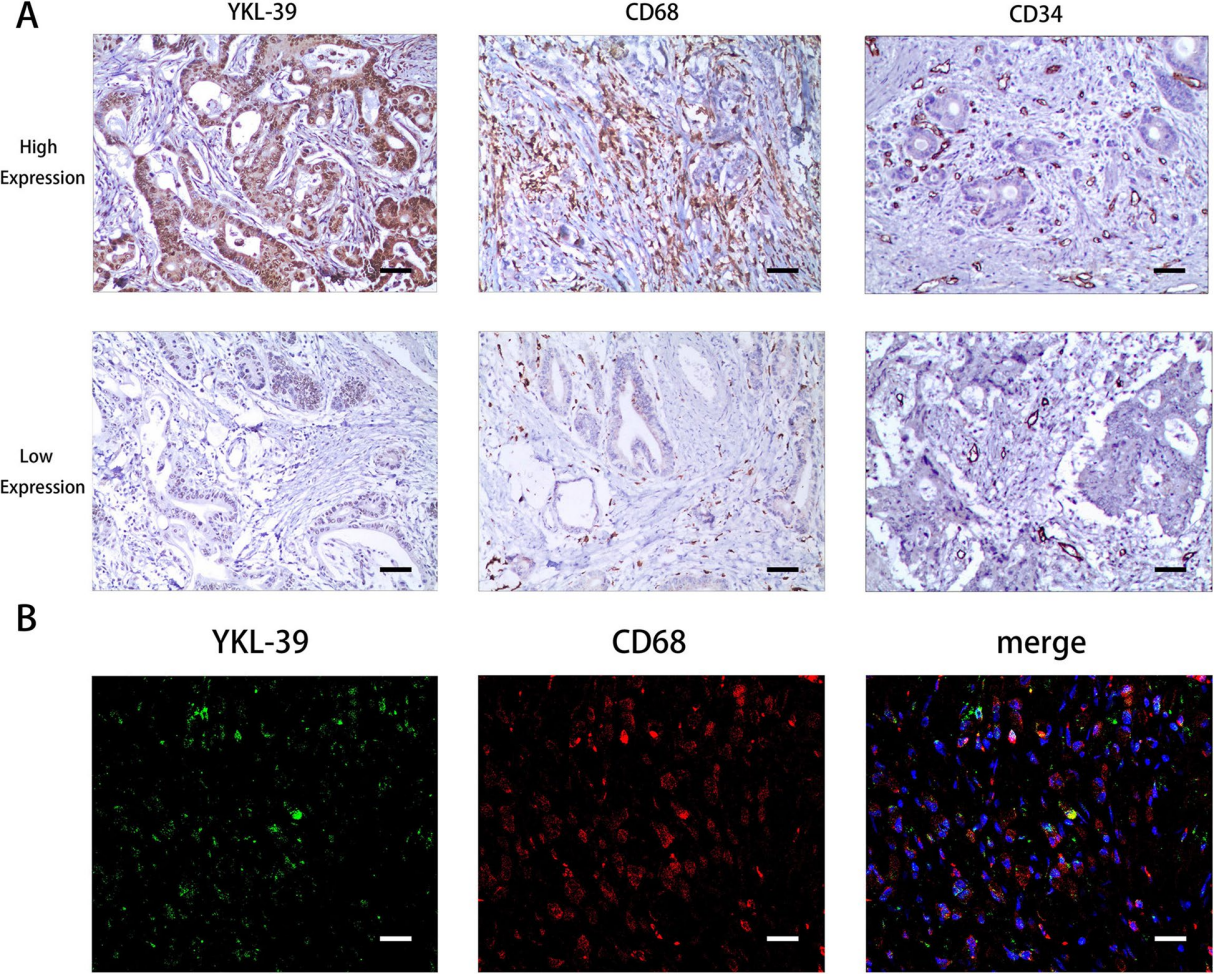
YKL-39, CD68 and CD34 IHC staining result and IF staining results. **A** IHC staining images of high and low expression of YKL-39, CD68, and CD34 protein in gastric adenocarcinoma. (× 200, horizontal lines represent 50 μm). **B** IF staining showed co-expression of YKL-39 (green fluorescence) and CD68 (red fluorescence) in gastric adenocarcinoma tissue (× 20, horizontal lines represent 500 μm)

IHC staining showed that YKL-39 was expressed in both the nucleus and cytoplasm of GC tumor cells and stromal cells (Fig. 1A). IHC staining for CD68 showed cytoplasmic staining, diffusely distributed in the tumor stromal cells, and was used to label TAMs (Fig. 1A). MVD is an important index of tumor angiogenic activity and intensity. To evaluate tumor angiogenesis, MVD was evaluated by IHC staining with CD34. To determine the relationship between YKL-39 protein expression and TAMs infiltration and MVD, we performed a correlation analysis with Spearman’s correlation coefficient (Table 2). The results show that positive reactions for both CD68 and CD34 were increased in the samples with higher YKL-39 expression. YKL-39 expression significantly correlates with CD68 and CD34 expression in GC (*p* < 0.001).

**Table 2:** Association of YKL-39, CD68, CD34 protein expression

		Total	YKL-39 expression			
			Low	High	*r*	*p* value
CD68	Low	51	38	13	0.666	< 0.001
	High	68	12	56		
					0.662	< 0.001
CD34	Low	60	42	18		
	High	59	8	51		

To determine the sites of expression of YKL-39 and CD68 in GC tissues, IF and confocal microscopy analysis was performed on samples from 10 patients with GC. The results show that YKL-39 was expressed in both tumor cells and tumor mesenchyme, and co-expressed with macrophages in the tumor mesenchyme (Fig. 1B).

### Survival analysis and the prognostic value of YKL-39, CD68 and CD34 expression in patients with GC

**Fig. 2:**
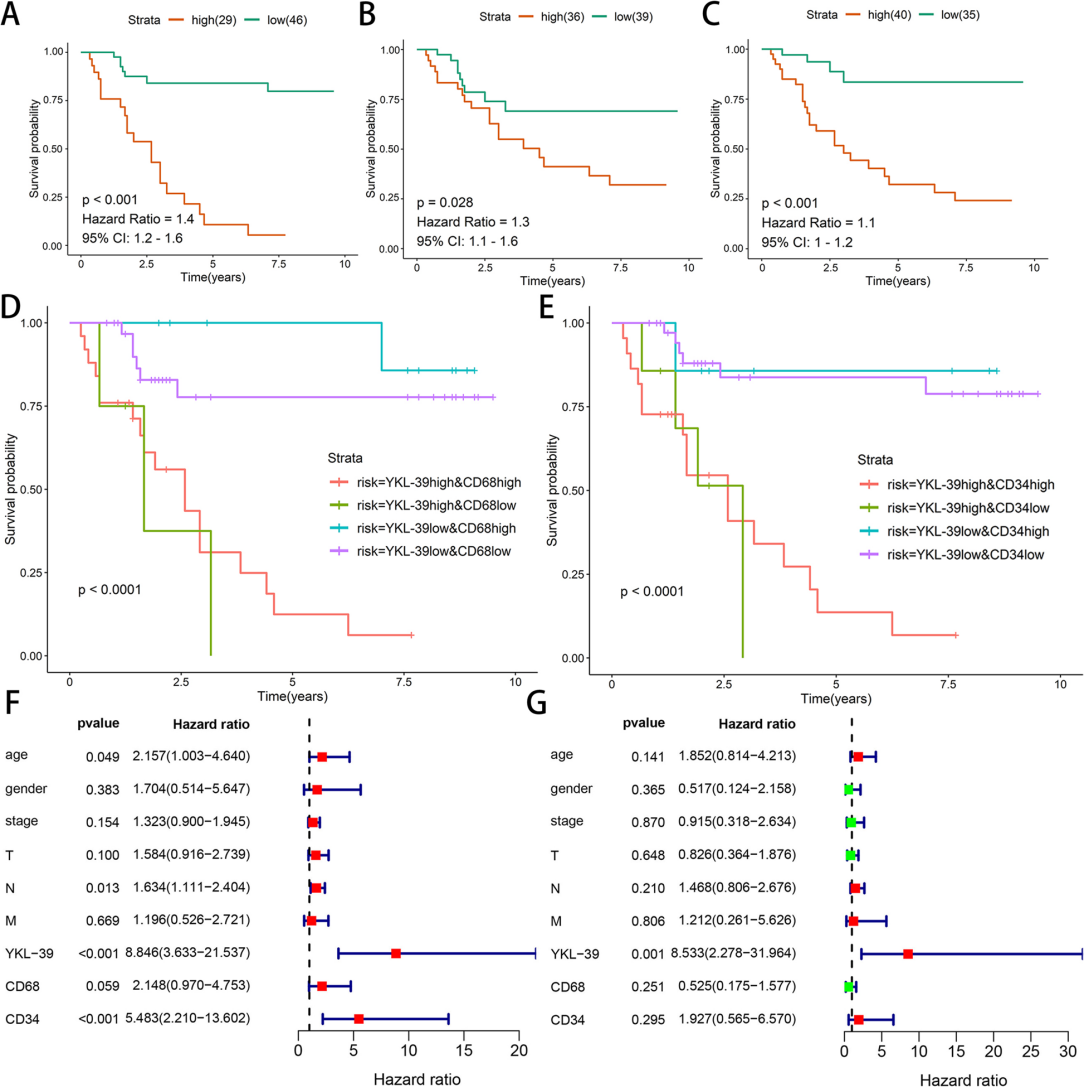
Survival analysis and the prognostic value of YKL-39, CD68 and CD34 expression in patients with GC. Kaplan–Meier survival curves of **A** YKL-39, **B** CD68, **C** CD34 expression in GC patients. **D** Kaplan-Meier survival curves of OS among the four patient groups stratified by YKL-39 and CD68 expression. **E** Kaplan-Meier survival curves of OS among four patient groups stratified by YKL-39 and CD34 expression. **F** Results of univariate COX regression analysis of YKL-39, CD68, CD34 expression, and clinicopathological information. **G** Results of multivariate COX regression analysis of YKL-39, CD68, CD34 expression, and clinicopathological information

Of the 119 cases included in this study, 75 were followed up and 44 were lost to follow-up, due to communication problems (change of phone number, refusal to answer). Among the 75 patients with follow-up, 28 patients had died during the last follow-up period. To explore the prognostic effects of YKL-39, CD68, and CD34 protein expression in GC, we plotted Kaplan-Meier survival curves and performed log-rank test (Fig. 2A–C). The results showed that patients with high YKL-39 expression had a significantly worse prognosis than those with low YKL-39 expression (*p* < 0.001, hazard ratio = 1.4). Patients expressing high CD68 and CD34 had a worse prognosis than those with low expression (*p* < 0.001).

As shown in Fig. 2D, when combining CD68 and YKL-39 to analyze prognosis, patients in the high CD68 low YKL-39 group had a significantly better prognosis than those in the high CD68 high YKL-39 group. The same situation also occurred when combining CD34 and YKL-39 to analyze the prognosis of patients (Fig. 2E) (*p* < 0.001). From Fig. 2D, patients in the high CD68-low YKL-39 group had the best prognosis. In contrast, as shown in Fig. 2E, the prognosis of patients in the high CD34-low YKL-39 group and low CD34-low YKL-39 groups did not differ significantly, indicating that CD34 expression did not have a significant impact on the prognosis of patients.

To further discern the independent prognostic role of YKL-39 in GC, we performed Cox regression analysis. The results of the univariate independent prognostic analysis showed a statistically significant relationship between age, N-stage, YKL-39 expression, MVD and survival outcome (*p* < 0.05) (Fig. 2F). Multivariate COX regression results showed that only YKL-39 expression was an independent prognostic factor for GC (*p* < 0.05) (Fig. 2G). Our results demonstrate that YKL-39 may serve as an independent prognostic factor for GC.

## Discussion

Despite advances in diagnostic and therapeutic techniques, GC retains a median survival of less than 12 months for patients with advanced GC [3]. Therefore, there is an urgent need to explore new biomarkers to predict the prognosis of GC patients as well as to guide individualized treatment. In this study we measured the expression of YKL-39, CD68, and CD34 in GC cells and mesenchyme by IHC, determined the distribution of YKL-39 and TAMs in GC tissue by IF, and analyzed the role of YKL-39 in GC. Our results indicate that YKL-39 an independent prognostic factor in GC.

Both chitinases and CLPs contain Glyco-18 structural domains, and in humans, chitinases include acidic mammalian chitinase and chitotriosidase, which cleave chitin polymers into oligosaccharides and release monosaccharides from the ends of chitin polymers [27]. CLPs do not possess glycosyl hydrolase activity due to the substitution of the catalytic glutamate in the active site DxxDxDxE motif terminus by leucine, isoleucine or tryptophan, but due to the Glyco-18 structural domain, they still have sugar-binding properties [28–30]. The human CLPs include YKL-40, YKL-39, and SI-CLP, of which YKL-40 (CHI3L1) is the best-studied and is upregulated in a variety of inflammatory and neurodegenerative diseases and cancers [16]. YKL-40 mediates activation of mitogen-activated protein kinases and phosphoinositide 3-kinase pathways through phosphorylation of extracellular signal-regulated kinase 1/2 and protein kinase B. Expression of YKL-40 is elevated in a variety of cancers, including breast cancer, colon cancer, lung cancer, prostate cancer, bladder cancer, and GC, and promotes tumor progression by promoting tumor cell proliferation, rapid invasion and migration, and angiogenesis [31]. Previous studies have confirmed that anti-YKL-40 treatment showed good anti-angiogenic effects in animal tumor models both in vivo and in vitro [32]. YKL-39 and YKL-40 show some similarity in size and amino acid sequence, which suggests that YKL-39 may have a similar biological function to YKL-40 [15]. Our results suggest that YKL-39 expression is associated with TAMs and angiogenesis in GC. As the most vital immune cells in the TME, the infiltration status of TAMs significantly affects the patient’s response to treatment and their prognosis [33, 34]. TAMs can stimulate solid tumor development and invasion by secreting a variety of pro-angiogenic factors, such as VEGF-A, PLGF and transforming growth factor-β [35]. The level of TAMs is positively correlated with the degree of MVD in a variety of tumors [11, 36]. In recent studies, YKL-39 has been identified in breast cancer as having monocyte chemotactic activity and pro-angiogenic activity, which is consistent with the functions of YKL-39 we found in GC. YKL-39 does not work directly on tumor cells but can influence tumor progression by altering the state of the TME, and elevated YKL-39 levels are associated with increased risk of metastasis and post-chemotherapy drug resistance in breast cancer [21]. Our results also show that higher YKL-39 protein expression in GC tissues was associated with deeper tumor infiltration, more lymph node metastases, and higher TNM stage. Similarly, high YKL-39 protein expression in renal cell carcinoma is significantly associated with tumor relapse, size, grade, and T-stage [22]. The Kaplan-Meier survival curve and log-rank test demonstrates that YKL-39 expression correlated with the prognosis of GC patients, and multivariate Cox regression analysis indicated that YKL-39 expression in tumors was an independent predictor of GC progression. These results argue that YKL-39 is a valuable prognostic biomarker for GC. In the study by Liu et al, YKL-39 was shown to be associated with tumor immune infiltration and affect the prognosis of patients with glioma, and high expression of YKL-39 is an independent adverse prognostic factor in glioma patients [20]. In renal cell carcinoma, high expression of YKL-39 is associated with poor patient prognosis and tumor recurrence [22]. In summary, combined with the findings on the function of the YKL-39 and its effect on prognosis in other tumors, we suggest that YKL-39 may act aggressively in malignant tumors. Our results demonstrate that YKL-39 is associated with TAMs infiltration and tumor angiogenesis in GC, and we suggest that YKL-39 has the potential to become a target for anti-TAMs aggregation and anti-tumor angiogenesis in GC. Our study indicates that the specific function of YKL-39 in GC needs to be further confirmed by cellular experiments.

## Conclusion

In summary, our results demonstrate that YKL-39 expression in GC is positively correlated with the depth of tumor infiltration, lymph node metastasis, TNM stage, TAMs infiltration and tumor angiogenesis, and is significantly associated with the prognosis of GC patients. Our results indicate that YKL-39 is associated with angiogenesis and is involved in the progression of GC, and that YKL-39 has the potential to be a novel prognostic biomarker and therapeutic target for GC.

## Data Availability

Not applicable.

## Abbreviations

GC: Gastric cancer
OS: Overall survival
VEGF-A: Vascular endothelial growth factor-A
PLGF: Placental growth factor
TAM: Tumor-associated macrophages
TME: Tumor microenvironment
TNFα: Tumor necrosis factor α
CLPs: Chitinase-like proteins
SI-CLP: Stabilin-1 interacting chitinase-like protein
IHC: Immunohistochemistry
IF: Immunofluorescence
FFPE: Formalin-fixed, paraffin-embedded
TMA: Tissue microarray
MVD: Micro vessel density
